# Emerging Roles of 5-Lipoxygenase Phosphorylation in Inflammation and Cell Death

**DOI:** 10.1155/2019/2749173

**Published:** 2019-11-29

**Authors:** Qian-Yi Sun, Hong-Hao Zhou, Xiao-Yuan Mao

**Affiliations:** ^1^Department of Clinical Pharmacology, Xiangya Hospital, Central South University, 87 Xiangya Road, Changsha 410008, China; ^2^Institute of Clinical Pharmacology, Central South University, Hunan Key Laboratory of Pharmacogenetics, 110 Xiangya Road, Changsha 410078, China; ^3^Engineering Research Center of Applied Technology of Pharmacogenomics, Ministry of Education, 110 Xiangya Road, Changsha 410078, China; ^4^National Clinical Research Center for Geriatric Disorders, 87 Xiangya Road, Changsha, 410008 Hunan, China

## Abstract

5-Lipoxygenase (ALOX5) is an iron-containing and nonheme dioxygenase that catalyzes the peroxidation of polyunsaturated fatty acids such as arachidonic acid. ALOX5 is the rate-limiting enzyme for the biosynthesis of leukotrienes, a family of proinflammatory lipid mediators derived from arachidonic acid. ALOX5 also make great contributions to mediating lipid peroxidation. In recent years, it has been discovered that ALOX5 plays a central role in cell death including apoptosis, pyroptosis, and ferroptosis, a newly discovered type of cell death. According to the previous studies, ALOX5 can regulate cell death in two ways: one is inflammation and the other is lipid peroxidation. Meanwhile, it has been shown that ALOX5 activity is regulated by several factors including protein phosphorylation, ALOX5-interactng protein, redox state, and metal ions such as iron and calcium. In this review, we aim to summarize the knowledge on the emerging roles of ALOX5 protein phosphorylation in the regulation of cell death and inflammation in order to explore a potential target for human diseases.

## 1. Introduction

Lipid peroxidation, which preferentially oxidizes polyunsaturated fatty acids (PUFAs), has been involved in the etiology of diverse pathological conditions including neurological disorders, cancers, diabetes, and cardiovascular disease [1–6]. Generally, PUFAs include arachidonic acid (AA), linoleic acid, and docosahexaenoic acids [7]. AA, one of the most important PUFAs in mammalian cells, is not only required for maintaining the membrane integrity but also serves as the direct precursor of many bioactive mediators such as leukotrienes (LTs), prostaglandins, thromboxane A2, epoxyeicosatrienoic acid, and endocannabinoids [8]. It is generally accepted that AA is metabolized by three sorts of enzymes: cyclooxygenases, cytochrome p450s, and lipoxygenases (ALOXs) [7, 9]. Among these enzymes, ALOXs make the biggest contribution to the generation of lipid peroxides [7]. A human ALOX family has six functional subtypes (ALOX5, ALOX12, ALOX12B, ALOX15, ALOX15B, and ALOXE3) while the mouse has seven different sorts of ALOX isoforms (Alox5, Alox15, Alox15b, Alox12, Alox12b, Aloxe3, and Alox12e) [10]. ALOX5 serves as a rate-limiting enzyme responsible for the biosynthesis of LTs which are the major mediators of inflammation, finally causing multiple human diseases including asthma, cancers, atherosclerosis, diabetes, and Alzheimer's disease [11–15]. The direct evidence showing a crucial role of ALOX5 in disease arises from the fact that ALOX5 overexpression aggravates memory deficits in a mouse model of Alzheimer's disease [16]. In contrast, deficient ALOX5 was previously found to promote cognitive recovery [16]. The specific function of ALOX5 is to catalyze AA into an unstable intermediate, 5-hydroperoxyeicosatetraenoic acid [17, 18]. 5-Hydroperoxyeicosatetraenoic acid can be metabolized by glutathione peroxidase into 5-hydroxyeicosatetranoic acid which is further converted into 5-oxo-eicosatetraenoic acid. ALOX5 also catalyzes 5-hydroperoxyeicosatetraenoic acid into LTA4, which can further generate proinflammatory factors including LTB4 and LTC4 by LTA4 hydrolase and LTC4 synthase, respectively [17, 18]. Additionally, ALOX5 is a crucial enzyme that mediates lipid peroxidation by producing lipid peroxides [7]. Excessive lipid peroxidation is easy to occur in phospholipids, the main components of plasma membrane, leading to membrane rupture and evoking cell death [7]. Activation of the cell death pathway can also promote inflammatory reactions by releasing damage-associated molecular patterns (DAMPs) [19]. Obviously, the activity of ALOX5 is rather essential for the reactions described above. The cellular enzymatic activity of ALOX5 is regulated by several factors including protein phosphorylation, metal ions (iron and calcium), substrate concentration, redox state, gene expression, and ALOX5-activating protein [8, 20–24]. Mounting evidence has revealed that the biological function of ALOX5 can be manipulated by protein phosphorylation, the most important posttranslational modification discovered in 1959 [25]. ALOX5 protein phosphorylation occupies a very essential position for ALOX5 translocating to nuclear membranes, which is necessary for ALOX5 activation [26]. Thus, we currently make a comprehensive review of the emerging roles of ALOX5 protein phosphorylation in inflammation and cell death in order to explore effectively the therapeutic targets for curing human diseases.

## 2. Overview of ALOX5 Phosphorylation

### 2.1. Brief Introduction of Effects on ALOX5 Activity

As described above, ALOX5 shows the great significance in the synthesis of LTs and makes contributions to human diseases. With the researches of ALOX5, many evidences have shown that some factors are crucial for the regulation of ALOX5 enzymatic activity. These factors include Ca^2+^, ATP, redox state, ALOX5-activating protein, gender difference, and phosphorylation [27–32]. Upon stimulation, the upregulation of intracellular Ca^2+^ could combine within the C2-like domain of ALOX5 and increase the nuclear translocation of ALOX5 [27]. ATP also stimulated ALOX5 directly and affected the production of lipoxins and LTs [28]. Since ALOX5 is a kind of iron enzyme, the redox cycle between ferrous and ferric forms is important absolutely and exogenous iron increases ALOX5 ability to combine with the nuclear membrane [29]. ALOX5-activating protein, as a regulatory protein, could transfer AA to ALOX5 effectively [30]. The distribution of ALOX5 is also related to cell types and regulated by androgens in human neutrophils and monocytes, so ALOX5 is connected with gender difference [31]. Absolutely, as a substrate for various protein kinases, ALOX5 itself contains multiple phosphorylation sites and disparate phosphorylation modulates its subcellular localization [32].

### 2.2. The Phosphorylation of ALOX5

ALOX5 has been reported to be phosphorylated by different kinases at several sites including Ser271, Ser663, Ser523, Tyr42, Tyr53, Tyr94, and Tyr445 (Table 1). Ser271, Ser663, and Ser523 are found to be phosphorylated by p38 MAPK-activated protein kinase 2 (MK2), extracellular signal-regulated kinase (ERK), and protein kinase A (PKA), respectively, while Tyr42, Tyr53, Tyr94, and Tyr445 are activated by Src kinase [33].

Different phosphorylation sites have different functions for ALOX5. It has been demonstrated that MK2 or ERK are identified to cause ALOX5 phosphorylation at Ser271 or Ser663 residue, enhancing ALOX5 activity while phosphorylation at Ser523 has an opposite effect [34]. Based on in-gel kinase assays, activated MK2 phosphorylates ALOX5 in human polymorphonuclear leukocytes and Mono Mac 6 (MM6) cells which lead to ALOX5 activation [34, 35]. Consistently, the activation described above by some stimulus could be blocked by SB203580 (a p38 MAPK inhibitor) and mutation of Ser271 to Ala [34, 36, 37]. Another phosphorylation site that promotes ALOX5 activation is ERK-dependent Ser663 phosphorylation [37]. In the presence of PUFAs, ALOX5 is a rather strong substrate for ERK and the phosphorylation site, Ser663, seems to be related to ALOX5 activation in MM6 cells and polymorphonuclear leukocytes [35, 38]. Correspondingly, S663A mutant inhibited the synthesis of ALOX5 products and the ERK inhibitor reduces the nuclear translocation of ALOX5 in human blood neutrophils [35, 38]. At the same time, there is a connection between Ser271 and Ser663. Firstly, phosphorylation at Ser271 and Ser663 is facilitated by PUFAs such as AA [39]. It was also previously reported that some stimulus such as chemotactic factors, proinflammatory cytokines, Ca^2+^-mobilizing agents, phorbol esters, and cell stresses (UV light, oxidative stress, osmotic shock chemical stress, heat shock, and genotoxic agents) gave rise to the activation of p38 MK2 and ERK and induced the ALOX5 translocation to the nucleus [39]. Moreover, the combination of two sites can enhance the synthesis of ALOX5 products when the level of intracellular Ca^2+^ is low particularly [33]. Besides, the phosphorylation of ALOX5 is also confirmed to be activated by tyrosine kinase *in vitro* and *in silico* methods [40]. Reversely, tyrosine inhibitors reduce the generation of 5-hydroxyeicosatetranoic acid in calcium ionophore-stimulated HL-60 cells and inhibit ALOX5 activity [41]. Unlike the several phosphorylation sites described above, the phosphorylation at Ser523 by PKA plays an opposite function. Activated PKA inhibits ALOX5 and subsequent generation of LTs via direct ALOX5 phosphorylation *in vivo* and *in vitro* [42]. The overexpression and pharmacological activation of PKA enhance ALOX5 phosphorylation at Ser523 but suppress ALOX5 activity [43]. In contrast, replacement of Ser523 with alanine or glutamate leads to increased activity of ALOX5 [42, 43]. These findings indicate that ALOX5 phosphorylation by PKA at Ser523 residue results in the inhibition of nuclear translocation and subsequent reduction of ALOX5 activity. In other words, ALOX5 phosphorylation is a key factor for ALOX5 activity.

## 3. Roles of ALOX5 Phosphorylation in Inflammation

**Figure 1:**
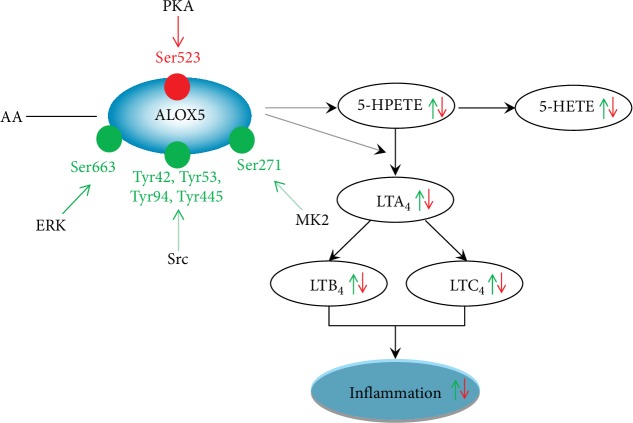
Role of ALOX5 protein phosphorylation in inflammation.

Inflammation is indispensable for human life. However, excessive release of inflammatory factors can induce hazardous consequences. Inflammatory mediators usually trigger the generation of inflammatory signs including the accumulation of leukocytes. LTs constitute one of the major types of inflammatory mediators, which are produced from AA. ALOX5 catalyzes the rate-limiting step of the LTs [17]. Many studies have demonstrated that ALOX5-mediated inflammation contributed a lot to diseases. It is reported that the recruitment of eosinophils in peritoneal cavity required ALOX5 and the blockage of ALOX5 inhibited inflammation and immunity [46]. ALOX5 also played a crucial role in inflammation-related cancers like colorectal cancer, breast cancer, and glioma [47–49]. Consequently, ALOX5 activity is vital for inflammatory reactions. At the same time, there is mounting evidence showing the significance of ALOX5 phosphorylation in the synthesis of LTs (as shown in Figure 1). For instance, phosphorylation at Ser271 and Ser663 residues was found to promote the ALOX5 nuclear translocation and facilitate the production of LTs [50]. On the contrary, ALOX5 Ser523 phosphorylation was previously reported to suppress ALOX5 activity and subsequently diminish LT generation [50, 51]. The protein phosphorylation of ALOX5 at Ser271 residue was also reported to be activated by MK2, leading to the nuclear translocation of ALOX5 and the stimulation of LT synthesis HEK293 cells [52]. Similarly, the upregulation of ALOX5 Ser663 phosphorylation can also induce LT production in human dendritic cells treated with lipopolysaccharide [53]. These findings suggest that MK2 and ERK are two major kinases responsible for enhancing ALOX5 phosphorylation and contributing to the biosynthesis of LTs. In terms of PKA, its activation has an opposite effect on ALOX5 activity. In detail, it was previously found that PKA activation caused enhanced ALOX5 protein phosphorylation at Ser523 residue but induced a low LT level in NIH3T3 cells [41]. Despite limited reports on the direct role of ALOX5 phosphorylation in human diseases, there is indirect evidence demonstrating that ERK activation exacerbates brain edema in a rat model of cerebral ischemia [54]. ALOX5 was upregulated in a rat model of focal cerebral ischemia while the inhibition of ALOX5 could accelerate functional recovery [55]. According to the description as mentioned above, ERK can activate ALOX5 protein phosphorylation at Ser663 residue. It indicates that ERK-mediated ALOX Ser663 phosphorylation results in brain ischemic impairment. Further investigation is essential to clarify this speculation. Additionally, atorvastatin, a common antiatherosclerotic drug was previously found to increase the level of ALOX5 phosphorylation at Ser523 (phosphorylated by PKA) which promoted the production of 15-epilipoxin-A4, an anti-inflammatory mediator [47], suggesting that the antiatherosclerotic potential of atorvastatin is related to suppressing ALOX5 activity induced by PKA-mediated ALOX5 Ser523 phosphorylation and inhibitory inflammation.

ALOX5 is the rate-limiting enzyme for the synthesis of LTs, which is the key mediator of inflammation. ALOX5 firstly catalyzes the conversion of AA into 5-hydroperoxyeicosatetraenoic acid and to LT4. The latter can be metabolized by LTA4 hydrolase and LTC4 synthase to generate LTB4 and LTC4, respectively. 5-Hydroperoxyeicosatetraenoic acid can also generate 5-hydroxyeicosatetranoic acid mediated by glutathione peroxidase. There are several phosphorylation sites of ALOX5. MK2 phosphorylates ALOX5 at Ser271 while ERK at Ser661. Src also stimulates ALOX5 activation and the phosphorylation sites contain Tyr42, Tyr53, Tyr94, and Tyr445. Ser523 phosphorylated by PKA shows a negative role on the synthesis of LTs.

## 4. Role of ALOX5 Phosphorylation in Cell Death

**Figure 2:**
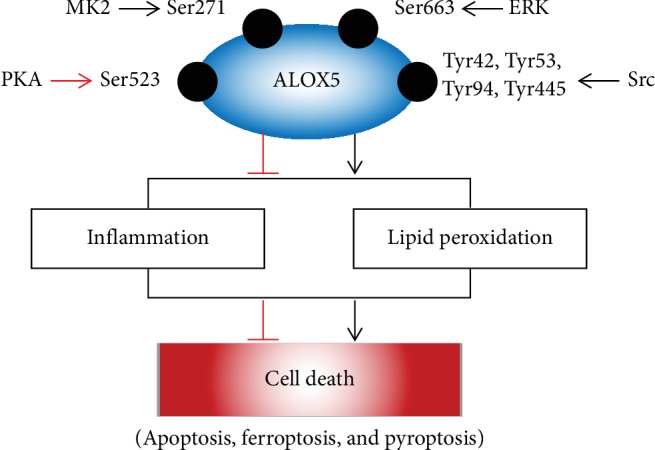
Role of ALOX5 protein phosphorylation in cell death.

Protein phosphorylation, the most studied posttranslational modification in cell life, is the most basic and important mechanism for regulating the activity of protein [25]. Due to its simplicity, reversibility, and flexibility, protein phosphorylation regulates the cell life in almost every aspects including cell death [25]. Our previous study shows that enhancement of GABA_A_R *γ*2 Ser327 phosphorylation was able to decrease neuronal apoptosis in an epileptic model and confirms the importance of protein phosphorylation in regulating cell death [56]. As is known to all, the function of ALOX5 needs the participation of protein phosphorylation; we can make a reasonable inference that protein phosphorylation may regulate cell death by affecting ALOX5 activity (Figure 2).

ALOX5 protein phosphorylation plays a central role in ALOX5, and the latter is widely studied in cell death. ALOX5 can affect cell death in two ways. On the one hand, cell death is the central feature of inflammation. The LTs, produced by ALOX5, are a group of proinflammatory mediators. On the other hand, ALOX5 itself is also a key enzyme in mediating lipid peroxidation which can lead to cell death such as apoptosis, ferroptosis, and pyroptosis.

Accumulative investigations have revealed that ALOX5 is able to affect cell death via two facets. On the one hand, cell death has been regarded as the central feature of inflammation [57]. It is well known that cell death in parenchymal cells can release a kind of “danger signals,” that is to say, DAMPs which include ATP, histones, nucleotides' high-mobility group protein B1, and cytokines, promote inflammation [58, 59]. In return, DAMPs can further evoke inflammation and cell death [49]. In other words, inflammation and cell death, which can affect each other, regulate organ homeostasis. Consistently, dysfunction of either aspect will lead to pathological conditions. In the same time, it was shown that the inhibition of ALOX5 decreased neuronal cell death by blocking inflammatory responses [60]. On the other hand, ALOX5 is a key enzyme for triggering lipid peroxidation. Lipid peroxidation is broadly defined as catalyzing the insertion of the hydroperoxy group into lipids containing carbon-carbon double bonds such as PUFAs in phospholipids which are responsible for the integrity of the plasma membrane [1]. Excessive lipid peroxidation leads to the activation of cell death pathways [7]. There is evidence showing that exposure with lipid peroxidation byproduct 4-hydroxynonenal induces neuronal cell death in human neuroblastoma SH-SY5Y cells [19]. Available evidences show that lipid peroxidation evokes multiple patterns of cell death including apoptosis, pyroptosis, and ferroptosis [54]. For instance, lipid peroxidation induced by trichloroethylene stimulates caspase-3/7 and causes apoptosis in a human extravillous trophoblast cell line [61]. Meanwhile, the downregulation of ALOX5 via RNA interference or an ALOX5 inhibitor could decrease apoptosis induced by carcinogen benzidine in the human tracheobronchial epithelial cell [62]. Pyroptosis, a kind of proinflammatory cell death regulated by a caspase family, is also related to lipid peroxidation. A recent study showed that knockout of glutathione peroxidase 4 increased lipid peroxidation which led to the cleavage of gasdermin D and subsequent pyroptosis in myeloid lineage cells [63]. More notably, ferroptosis is a newly cell death discovered by Stockwell in 2012 [64]. Lipid peroxidation is a critical feature of ferroptotic cell death [64]. It is involved in various diseases such as neurological disorders, renal failure, and cancers. For instance, the inhibition of ferroptosis had a neuroprotective effect on a FeCl_3_-induced epileptic model [65]. Many researches have shown that ALOX5 functions as a target for ferroptosis. Pharmacological inhibition of ALOX5 by zileuton exhibited a neuroprotective role in glutamate-induced HT22 cells by blunting ferroptosis [66]. Consistently, it was also previously confirmed that the inhibition of ALOX5 protected neurons from ferroptotic death in mice with hemorrhagic stroke via neutralizing lipid peroxides [67]. And that, ferroptosis is also a type of inflammation-associated cell death. As a typical necrosis, ferroptosis triggers the release of DAMPs and further causes ferroptosis in renal failure by decreasing inflammation produced by ALOX5 [68]. Activation of the ferroptotic process by RAS-selective lethal 3, erastin, FIN56, or sorafenib can induce the release of high-mobility group protein B1, a proinflammatory cytokine [69]. Moreover, the significance of ALOX5 in ferroptosis also arises from the fact that ALOX5 is an iron-containing enzyme and iron accumulation often occurs in ferroptosis. Given that protein phosphorylation is vital for ALOX5 activity, ALOX5 protein phosphorylation may be involved in the ferroptotic process. Further investigation is essential to clarify this item. There was a report that ALOX5 activation was independent with calcium upregulation (a significant ALOX5 induction factor) because calcium chelators failed to stop ferroptosis [70]. However, ALOX5 activation requires ALOX5 nuclear localization and the latter is controlled by Ca^2+^ levels and several kinases [71]. In addition, it was also reported the activation and ERK participated in glutamate-induced cell death in HT22 neurons [72]. Further study indicated that the ablation of glutathione peroxidase 4 could induce ERK activation and elevate inflammation which led to spinal motor neuron degeneration featured with ferroptosis [73]. These results suggest the importance of the ERK signaling pathway in ferroptosis. Collectively, ALOX5 phosphorylation mediated by ERK signaling may be a target for ferroptosis.

## 5. Concluding Remarks and Future Directions

Inflammation and cell death are two critical steps in human life. Under normal circumstances, moderate inflammatory reaction and cell death are beneficial. However, excessive inflammation and abnormal activation of the cell death pathway often result in harmful consequences, finally leading to the pathogenesis of various human diseases. As mentioned above, ALOX5 has been extensively found to activate inflammatory reactions and trigger various cell death modes including apoptosis, ferroptosis, and pyroptosis. And protein phosphorylation is vital for the modulation of ALOX5 activity. Therefore, deciphering the molecular mechanism how protein phosphorylation affects ALOX5 activity would be of utmost importance to the regulation of inflammation and cell death. Up to date, there are limited publications on drugs targeting ALOX5 protein phosphorylation for curing human diseases which involve excessive inflammation and deregulated cell death pathways. Extensive investigations have revealed that kinases which modulate ALOX5 phosphorylation serve as therapeutic targets for treating diseases. The therapeutic potential of kinase inhibitors are summarized in Table 2. It can be speculated that these compounds may function in various diseases by regulating ALOX5 phosphorylation. Meanwhile, there are abundant reports about the role of these kinases (MK2, ERK, PKA, and Src) in diseases. MK2 inhibitor MK2i improved the cornea wound healing and reduced corneal inflammation induced by alkali burn in rats [74]. PHA781089, another MK2 inhibitor, could induce apoptosis in hepatocellular carcinoma cell lines [75]. These facts implicate that ALOX5 phosphorylation via MK2 inhibition may be an attractive target for treating human diseases. Similar to p38 MAPK/MK2 cascade, the MAPK kinase/ERK pathway also is a vital target for human disease [76]. The MAPK kinase/ERK inhibitor PD98059 could reduce focal infarct volume in a focal cerebral ischemia model [77]. Another report identified that MAPK kinase/ERK inhibitor U0126 blocked tumor growth of embryonal rhabdomyosarcoma both *in vitro* and *in vivo* [78]. PKA is also a crucial kinase for ALOX5 phosphorylation but share an opposite function for ALOX5. PKA activator 8-Br-cAMP had the ability to inhibit cell proliferation of human breast cancer [79]. In addition to these specific kinase inhibitors/activators, there are other compounds have shown the ability to improve human diseases by affecting these kinase pathways. MK886 blocked the ERK/ALOX5 pathway to induce apoptosis in human gastric cancer cells [80]. It was reported that MAG-EPA suppressed lipopolysaccharide-induced inflammation in human peripheral blood mononuclear cells via inhibition of p38 MAPK phosphorylation [81]. Based on these evidences, we can make a speculation that ALOX5 phosphorylation monitored by kinase signaling pathways maybe a therapeutic target for human diseases. Future investigations are indispensable for clarifying the roles of kinase-mediated ALOX5 phosphorylation in the regulation of inflammation and cell death.

## Figures and Tables

**Table 1:** Phosphorylation sites on ALOX5 and corresponding kinases and effects.

Phosphorylation sites	Kinases	Effect	References
Ser271	MK2	ALOX5 activation	[36, 37]
Ser663	ERK	ALOX5 activation	[35, 44]
Ser523	PKA	ALOX5 inactivation	[44, 45]
Tyr42, Tyr53, Tyr94, and Tyr445	Src kinases	ALOX5 activation	[40, 41]

**Table 2:** Therapeutic potential of ALOX5 phosphorylation-associated kinases in human diseases.

Compound	Kinases	Effects	Results	References
PD98059	MEK	ERK inhibition	Decreased focal infarct volume in a focal cerebral ischemia model of rats	[77]
			Decreased infarct area after ischemic preconditioning in the porcine heart	[82]
U0126	MEK	ERK inhibition	Reduced tumor growth in the embryonal rhabdomyosarcoma cell	[78]
			Reduced tumor mass with the DNA protein kinase catalytic subunit and enhanced radiosensitivity of rhabdomyosarcoma cells	[83]
			Inhibited ferroptosis in HT-1080, BJeLR, and Calu-1 cells	[64]
			Decreased infarct area after ischemic preconditioning in the porcine heart	[82]
			Inhibited the damage induced by oxygen deprivation and nitric oxide toxicity in mouse primary neurons and reduced infarct volume in a rat ischemia model	[84]
SCH772984	ERK1/2	ERK inhibition	Induced cell apoptosis in NRAS mutant, BRAF mutant, and wild-type melanoma cells	[85]
PLX4032	RAF	ERK inhibition	Inhibited tumor proliferation in BRAFV600E cells	[86]
PD0325901	MEK	ERK inhibition	Antitumor activity in patients with advanced melanoma	[87]
SL327	MEK1/2	ERK inhibition	Altered the exocytosis machinery and decreased audiogenic seizure audio-stimulated rats via inhibition of MEK/ERK cascade	[88]
PF3644022	MK2	MK2 inhibition	Reduced the synthesis of proinflammatory mediators and tumor volume in murine colorectal cancer cells and eliminated tumor development in the azoxymethane/dextran sodium sulfate model of colitis-associated colorectal cancer	[89]
			Induced apoptosis in 5 hepatocellular carcinoma cell lines including HepG2, Huh7, Hep3B, HLE, and HLF	[75]
PHA781089	MK2	MK2 inhibition	Induced apoptosis in 5 hepatocellular carcinoma cell lines including HepG2, Huh7, Hep3B, HLE, and HLF	[75]
MK2i	MK2	MK2 inhibition	Inhibited corneal inflammation induced by alkali burn in rats	[74]
CMPD1	MK2	MK2 inhibition	Induced apoptosis and blocked G2/M cell cycle in human MKN-45 and SGC7901 gastric cancer cells	[90]
8-Br-cAMP	PKA	PKA activation	Reduced tumor-initiating ability mesenchymal-to-epithelial transition of mesenchymal human mammary epithelial cells	[91]
			Inhibited LPS-induced expression of proinflammatory factors induced in human macrophages	[92]
			Decreased cell proliferation in human MDA-MB-231 breast cancer cells	[79]
PP2	Src	Src inhibition	Reduced epithelial-mesenchymal transition and tumor migration/invasion in breast cancer cells with a high vimentin level	[93]
			Inhibited tumor migration in canine mammary carcinoma cells	[94]
Src I1	Src	Src inhibition	Inhibited tumor migration in canine mammary carcinoma cells	[94]
LY-1816	Src	Src inhibition	Inhibited tumor proliferation, migration, and invasion and induced apoptosis in human pancreatic ductal adenocarcinoma	[95]
